# Comparison of safety and efficacy of liberal versus restrictive red blood cell transfusion thresholds on the quality of life in patients with myelodysplastic syndromes: a systematic review and meta-analysis

**DOI:** 10.1007/s00277-026-06789-5

**Published:** 2026-01-26

**Authors:** Saikat Mandal, Arkadeep Dhali, Suhasini Sil, Manideepa Maji, Joyisa Deb, Aswin K. Mohan, Suvro Sankha Datta

**Affiliations:** 1 Translational Medical Sciences, School of Medicine, University of Nottingham, Nottingham, England; 2 Sheffield Teaching Hospitals NHS Foundation Trust, Sheffield, UK, University of Sheffield, Sheffield, UK University of Edinburgh, Edinburgh, UK; 3 All India Institute of Medical Sciences, New Delhi, India; 4 Hull York Medical School, University of Hull, Hull, UK Haematology, Hull University Teaching Hospitals NHS Trust, Hull, UK; 5 All India Institute of Medical Sciences, Guwahati, India; 6 All India Institute of Medical Sciences, Bibinagar, India; 7 Tata Medical Centre, Kolkata, India

**Keywords:** Myelodysplastic syndromes, MDS, Liberal versus restrictive, Blood transfusion, Quality of life, Transfusion

## Abstract

Myelodysplastic syndromes (MDS) are clonal stem cell disorders managed by risk stratification: lower-risk disease receives erythropoiesis-stimulating agents; higher-risk disease receives azacitidine. Red blood cell (RBC) transfusions manage symptomatic anaemia and improve quality of life (QoL) but carry risks of iron overload and alloimmunisation. No standardised transfusion strategy exists, requiring systematic evidence synthesis comparing liberal versus restrictive haemoglobin (Hb) thresholds for their effects on quality of life and transfusion-related complications. We performed a systematic review evaluating liberal versus restrictive RBC transfusion thresholds in adults with MDS not undergoing curative treatment such as stem cell transplantation. Primary outcome was health-related QoL measured by validated instruments. Secondary outcomes included mortality, transfusion reaction, iron overload, RBC utilisation and rise in ferritin level. Of 4,295 records screened, 212 articles underwent full-text review, with three RCTs meeting inclusion criteria. Liberal transfusion strategies were associated with improved QoL compared to restrictive approaches, with pooled standardised mean difference (Hedges g) of 0.54 (95% CI 0.06–1.02; *p* = 0.33; I²=9.7%). Subgroup analysis of EQ‑5D outcomes across studies revealed minimal but statistically significant difference between strategies (pooled mean difference 0.084; 95% CI: 0.033 to 0.134). Mortality (hazard ratio 0.913; 95% CI 0.167–4.98) and transfusion reactions (risk difference − 0.01; 95% CI-0.10 to 0.09) did not differ. Notably, liberal thresholds required average of 4 additional RBC units per patient (95% CI 1.43–6.79), raising concerns about iron overload (rise in ferritin; mean difference 868 µg/L; 95% CI 482–1255). Limited evidence suggests that liberal transfusion strategies in MDS may improve short-term QoL but at the cost of increased transfusion burden, with no clear impact on survival or safety. A patient-centred, multidisciplinary approach remains essential when tailoring transfusion thresholds.

The study protocol has been registered in PROSPERO (CRD420251085221). https://www.crd.york.ac.uk/PROSPERO/view/CRD420251085221.

**Supplementary information:**

The online version contains supplementary material available at 10.1007/s00277-026-06789-5.

## Introduction

Myelodysplastic syndromes (MDS) are clonal disorders of haematopoietic stem cells characterised by bone marrow dysplasia and ineffective haematopoiesis. The resulting cytopenias including anaemia, neutropenia, and thrombocytopenia are associated with impaired quality of life. Anaemia commonly manifests with fatigue, pallor, and shortness of breath, while neutropenia increases the risk of recurrent or severe infections, and thrombocytopenia may result in easy bruising, petechiae, gingival bleeding, or epistaxis [1].

Prognostic classification is commonly based on the International Prognostic Scoring System (IPSS), the revised IPSS-R and International Prognostic Scoring System-Molecular (IPSS-M) [2]. Management plans rely heavily on this risk stratification. Guidelines from the British Society for Haematology [3] and the European Society for Medical Oncology [4] recommend a risk-stratified approach to managing symptomatic anaemia. For patients with low to intermediate-1 disease (IPSS system) or very low to intermediate (IPSS-R system), the initial management strategy predicts the likelihood of erythropoiesis-stimulating agent (ESA) with or without granulocyte-colony stimulating factor (G-CSF) response using the Nordic scoring model based on serum erythropoietin level and transfusion burden. Patients predicted to respond favourably should receive ESAs with or without granulocyte-colony stimulating factor (G-CSF), or erythroid maturation agents such as luspatercept. For patients with a low probability of ESA response, red cell transfusions should be initiated as first-line supportive treatment for symptomatic anaemia.

In selected cases, such as hypoplastic MDS, immunosuppressive therapy with antithymocyte globulin and ciclosporin may be considered [5]. Curative options like allogeneic haematopoietic stem cell transplantation (HSCT) are generally reserved for patients with intermediate-2 or high-risk disease. For older or comorbid patients not eligible for HSCT, transfusion support remains central to care [3–5]. The Swedish MDS register [6] mentions that around 50% patients were transfusion dependent at diagnosis.

Blood transfusions improve fatigue, pallor, and bleeding symptoms in patients with MDS. RBC transfusion strategies are typically categorised as either liberal or restrictive. A liberal strategy applies a higher haemoglobin threshold to maintain higher levels, while a restrictive strategy adopts a lower threshold and seeks to minimise transfusion exposure and associated risks [7, 8].

Low Hb level has been found to be associated with inferior quality of life among MDS patients and more specifically severity of anaemia has significant impact on quality of life of MDS patients [9–11]. Maintaining higher haemoglobin through liberal transfusion may improve quality of life but also increases the risk of all the early and delayed adverse effects of blood transfusion. Following liberal transfusion strategy enhances the risk of transfusion-related reactions such as haemolytic reaction, transfusion related acute lung injury, circulatory overload. Allogenic blood transfusion also enhances the risk of viral transmission such as HIV, hepatitis B, hepatitis C, Cytomegalovirus, Epstein–Barr virus and bacterial infections. Exposure to higher allogenic blood may cause iron overload, alloimmunisation [12].

Currently, there are no recommended clear transfusion strategies for patients with MDS. BSH guidelines suggest that clinicians may adopt individualised approaches, using tailored Hb thresholds for targeted symptom management of MDS patients [3]. However, the absence of standardised transfusion strategy creates a clinical dilemma, leading to variability in practice and uncertainty in optimal patient care. To date, no systematic review has comprehensively synthesised evidence comparing liberal versus restrictive transfusion thresholds for their impact on quality of life and transfusion-associated risks among MDS patients. A previous attempt at a systematic review in 2015 was limited by the absence of studies reporting quality-of-life outcomes [13]. To address this gap, we want to conduct an updated systematic review and meta-analysis. A preliminary feasibility search has confirmed the availability of relevant data for synthesis.

### Review objectives

This systematic review aims to assess the safety and efficacy of a liberal versus restrictive red blood cell transfusion threshold for managing better quality of life for patients with myelodysplastic syndrome (MDS) who are not receiving potentially curative treatment.

## Methods

This systematic review and meta-analysis were conducted in accordance with the Preferred Reporting Items for Systematic Reviews and Meta-Analysis (PRISMA) guidelines [13]. We included randomised controlled trials (RCTs) and observational studies comparing restrictive versus liberal RBC transfusion thresholds in MDS patients. Case reports and single-arm studies were excluded. Studies were eligible for inclusion regardless of language, publication status, or year of publication.

### Participants

Adults (≥ 18 years) with MDS receiving intermittent or regular RBC transfusions for supportive care and not undergoing curative therapy (e.g. HSCT) were included. All MDS subtypes and risk categories (WHO, IPSS, IPSS-R, IPSS-M) were eligible [2]. Studies limited to paediatric populations or other hematologic disorders were excluded unless adult MDS results were reported separately.

### Interventions

All the studies that compared threshold-based transfusion policies (restrictive versus liberal), accepting authors’ threshold definitions were included. For the purposes of this review, a restrictive transfusion threshold is one where patients receive transfusions only when haemoglobin (or haematocrit) falls below a specified lower trigger level, whereas a liberal transfusion threshold allows transfusion at a higher haemoglobin level (for example, a restrictive policy might use a transfusion threshold of 7–8 g/dL or 70–80 g/L, and for liberal policy a transfusion threshold of 9–10 g/dL or 90–100 g/L). The exact haemoglobin cut-off values defining “restrictive” and “liberal” policies may vary across studies. Co-interventions (e.g., iron chelation, ESAs, erythroid maturation agents) were permitted if balanced across groups and the transfusion threshold policy is the primary difference. Comparisons of transfusion versus no transfusion or unclear target policies were excluded.

### Outcomes and timing

Outcomes were evaluated as reported in the studies; Health-related quality of life measured with validated patient-reported instruments (e.g., EQ-5D, FACT-An, EORTC QLQ-C30) was considered the primary outcome in this study. Overall, composite scores were extracted at available study reported time points. Secondary outcomes included all-cause transfusion-related adverse events; RBC utilization (total units per patient, frequency of transfusion episodes, and transfusion intervals); iron overload as defined by study criteria. These outcomes were not used to determine eligibility but formed the basis for data extraction and analysis.

### Information sources and search strategy

MEDLINE, EMBASE, the COCHRANE registry of clinical trials (CENTRAL), Transfusion Evidence Library, and CINAHL electronic databases were searched without language restriction from inception to the search date (August 8, 2025). The search combined terms for myelodysplastic syndromes, transfusion, and transfusion strategies are attached in Appendix 1. We additionally screened reference lists, conference proceedings, trial registries, regulatory websites, and thesis repositories, and contacted authors for unpublished data.

**Fig. 1:**
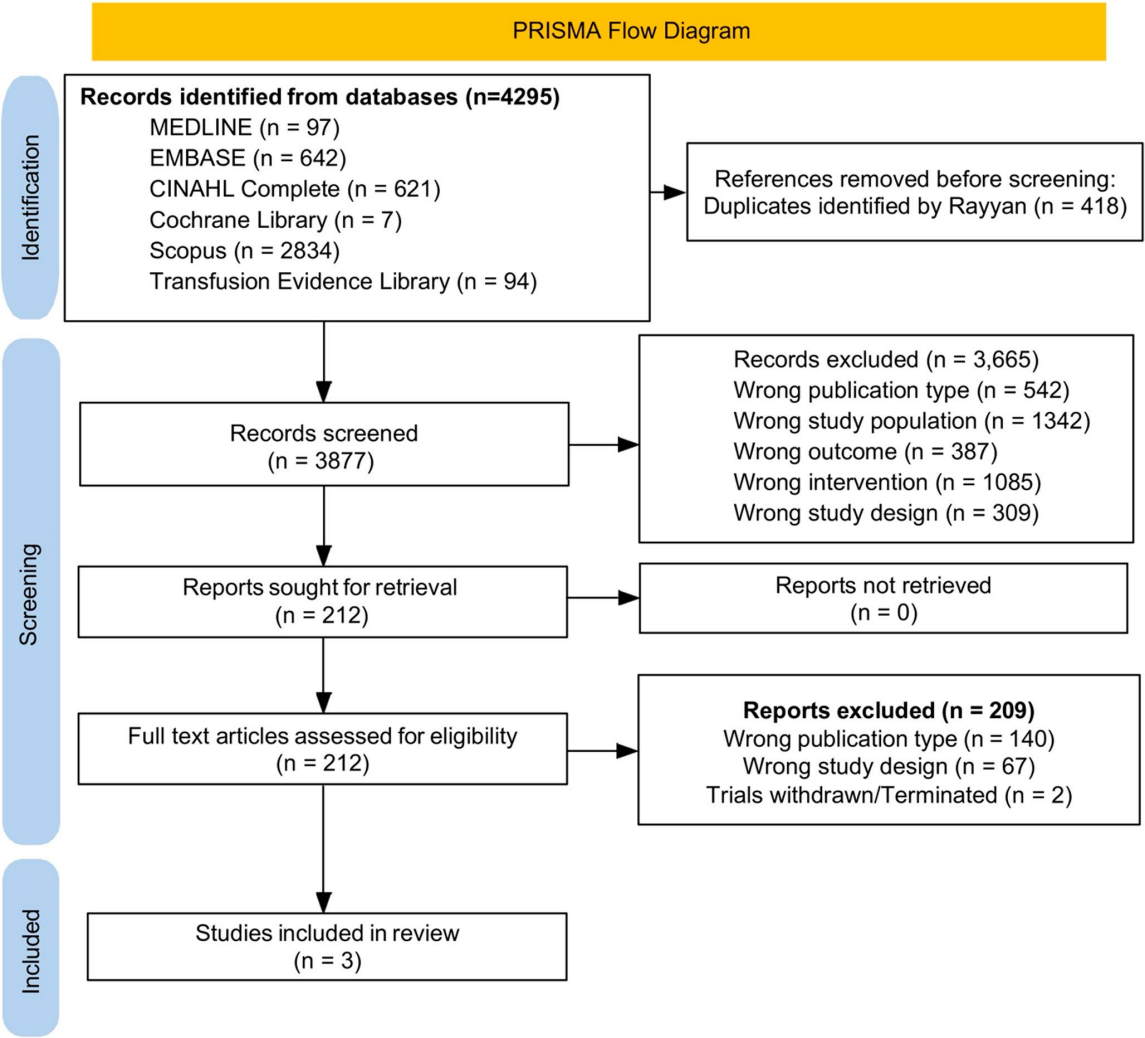
PRISMA flow diagram [16] detailing study selection process for systematic review, including identification, screening, and inclusion phases

A two-stage screening process was followed as per Cochrane and PRISMA guidelines [14, 15]. Two reviewers independently screened titles/abstracts and then assessed full texts using a standardized eligibility checklist; disagreements were resolved by discussion and, if necessary, adjudication by a third reviewer. Reasons for stage 1 and full-text exclusion were recorded, and the selection process was summarised in a PRISMA flow diagram [16] (Fig. 1).

Data were extracted independently by two reviewers using a piloted, standardized form. Data was entered into the Review Manager (Rayyan) and cross-checked by a second reviewer.

**Fig. 2:**
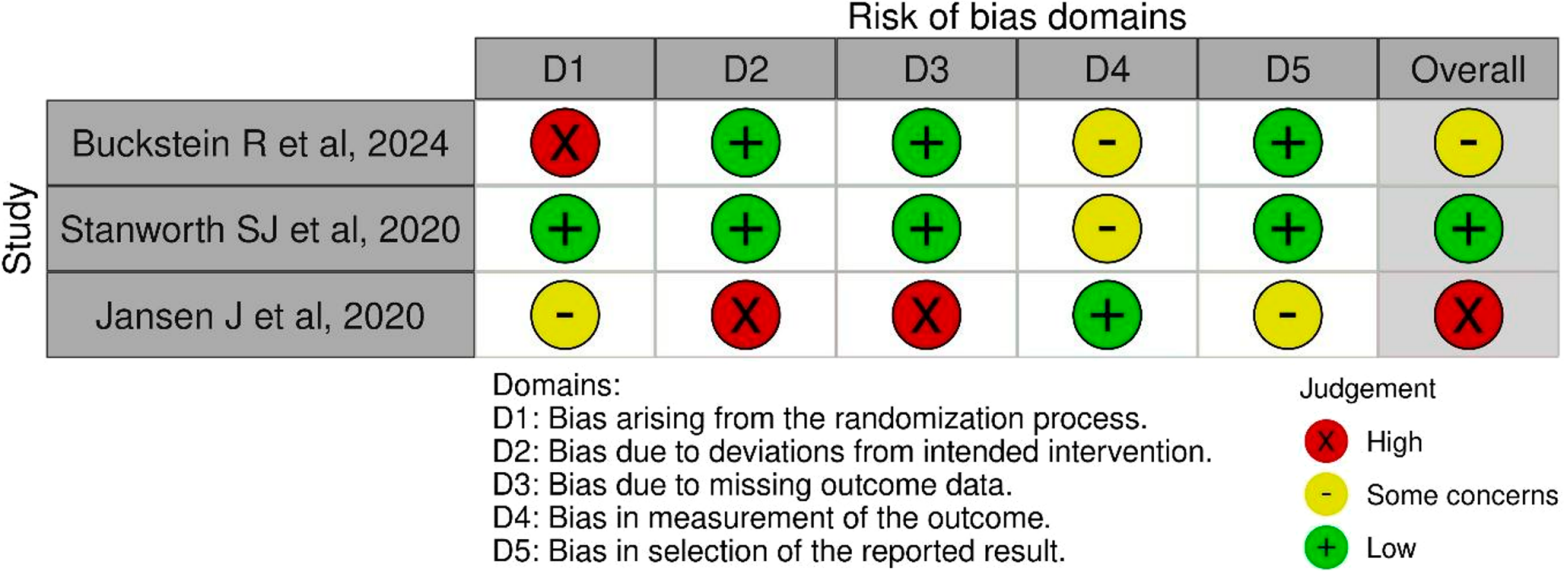
Risk of bias assessment for included studies [21–23] across five domains using standard color-coded judgments [17]

Two review authors assessed the risk of bias independently using established Cochrane tools, Risk of Bias 2 (RoB 2 tool) [17] (Fig. 2). The independent assessment by 2 reviewers were compared and any differences were resolved by discussion. A third reviewer was consulted upon nonresolution of differences. The results of risk of bias assessment were presented as a table and incorporated into results.

### Measures of treatment effect

For the liberal vs. restrictive transfusion threshold comparison, summary effect measures were selected by outcome type. Dichotomous outcomes were pooled as risk ratios (RR) using Mantel–Haenszel methods; when events were rare and several arms had zero events, we instead pooled risk differences (RD) to retain double-zero studies, applying a small continuity correction where required. Continuous outcomes were pooled as mean differences (MD) when the same scale was used and as standardised mean differences (SMD; Hedges’ g) when studies used different instruments or scales (e.g., EQ-5D index/SAUC vs. VAS). Time-to-event outcomes (e.g., overall mortality) were synthesised as hazard ratios (HR) using the generic inverse-variance method; when HRs and standard errors were not directly reported, we approximated them from available data following established approaches [18, 19].

Where appropriate, absolute effects and NNT/NNH were derived from pooled estimates and representative baseline risks and are reported where interpretable. For QoL, we harmonised to the ~ 3-month (≈ 12-week) timepoint and pooled effects as Hedges’ g (SMD) to account for differing instruments.

### Dealing with missing data

Study authors were contacted for missing outcomes. Intention-to-treat denominators were used when available. For dichotomous outcomes, participants lost to follow-up were treated as non-events unless study authors specified otherwise. For continuous outcomes, we used study reported per-protocol/complete-case summaries when ITT means/SDs were unavailable. When only medians and IQRs were reported, these were converted to means and SDs using standard formulae; when SDs were missing, they were imputed from available statistics. For the ferritin rise outcome, arm-level SDs for one trial were borrowed from the otherdue to non-reporting; this imputation is declared in the Fig. 9 legend and tested in sensitivity analyses. No other missing data were imputed beyond these standard conversions/imputations.

### Assessment of heterogeneity

Clinical and methodological heterogeneity were assessed qualitatively. Statistical heterogeneity was assessed with the χ² test and I², and τ² was estimated using the DerSimonian–Laird (DL) method. Given anticipated between-study differences, the primary model was random-effects (DL); fixed-effect models were used in sensitivity analyses. When heterogeneity was very high and unexplained, we provided a descriptive synthesis and explored potential sources via pre-specified subgroup analyses.

### Data synthesis

Meta-analyses were performed in R using the meta package [20].

Dichotomous outcomes: Mantel–Haenszel RR with 95% CIs; for rare events with zero-event arms, RD was used to include double-zero trials.

Continuous outcomes: Inverse-variance MD or Hedges’ g (SMD) with small-sample correction.

**Fig. 3:**
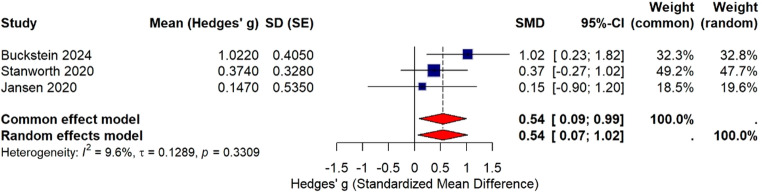
Forest plot showing the standardized mean difference (Hedges’ g) in quality of life scores for three studies (Buckstein 2024, Stanworth 2020, Jansen 2020) [21–23] comparing liberal versus restrictive transfusion strategies. For each study, mean (Hedges’ g), standard error (SE), individual 95% confidence interval, and study weights are displayed. The plot presents both common (fixed) and random effects models as pooled estimates. Summary diamonds indicate the overall effects for each model. Measures of heterogeneity (I² = 9.6%, τ = 0.129, *p* = 0.33) reveal low between-study variance

Time-to-event outcomes: Generic inverse-variance pooling of HRs (observed or approximated). Primary analyses used random-effects (DL); fixed-effect models were run for sensitivity. All tests were two-sided with P<0.05. Analyses are mentioned in results section figure 3 to figure 9. For datasets reporting medians/IQR only, we converted to means/SDs wherever feasible; narrative synthesis was reserved for outcomes not convertible. Double-zero trials were retained in RD analyses but would have been excluded from RR/OR models.

A Summary of Findings table was prepared using GRADE (Fig. 10). Outcomes included quality of life, all-cause mortality, serious transfusion-related adverse events, and transfusion requirements. Any downgrading/upgrading is footnoted per GRADE guidance.

### Subgroup analysis and investigation of heterogeneity

We performed exploratory, underpowered subgroup analyses:

1. Transfusion threshold level (e.g., restrictive trigger 7 vs. 8 g/dL; or magnitude of separation between policies).
2. QoL instrument (EQ-5D index/SAUC vs. VAS).

Subgroup differences were examined via χ² tests for interaction within stratified meta-analyses.

### Sensitivity analysis

We (i) compared fixed-effect vs. random-effects models; (ii) repeated analyses excluding high risk-of-bias or high attrition studies; (iii) re-ran continuous-outcome meta-analyses using alternative SD assumptions/conversions (including removing the borrowed SD for ferritin) to assess robustness of conclusions.

## Results

After screening 4295 RCTs and observational studies only 3 RCTs were found eligible for inclusion [21–23] (Table 1). Details of the screening process are mentioned in Fig. 1.

**Table 1:** Systematic review comparing Liberal and restrictive RBC transfusion approaches in MDS patients for patient reported quality of life score (Hedges g)

Study	QoL Instrument	*n* (L)	*n* (*R*)	Hedges g	SE	95% CI	Weight% (RE)	Follow up period (months)
Buckstein 2024 (RBC‑ENHANCE) (21)	EQ‑5D‑3 L (pre‑transfusion mean)	15	13	1.022	0.405	0.229 to 1.815	32.9	3
Stanworth 2020 (REDDS) (22)	EQ‑5D‑5 L (SAUC, median→mean; IQR→SD) *	18	20	0.374	0.328	−0.268 to 1.017	47.5	3
Jansen 2020 (TEMPLE) (23)	EuroQol VAS (0–100) at ~ 3 months	7	7	0.147	0.535	−0.902 to 1.196	19.6	3

Abbreviations: L, liberal; R, restrictive; SE, standard error; DL, DerSimonian–Laird

### I) Analysis of primary outcome (QoL)

 A random-effects meta-analysis (using Hedges’ g for standardized mean difference) was performed on quality-of-life scores, as studies employed different validated instruments (EQ-5D index vs. EQ-5D SAUC vs. VAS) with varying metrics. Pooling results through standardized effect sizes allowed meaningful synthesis despite measurement heterogeneity.

#### Assessment of reporting biases

All study effect sizes favour the liberal transfusion strategy, with Buckstein 2024 [21] showing the largest benefit. The pooled effect under both models suggests a positive standardized mean difference (SMD = 0.54), with the fixed effect confidence interval [0.09, 0.99] excluding zero (statistically significant), while the random effects interval [0.07, 1.02] also suggests significance (Fig. 3) but accounts for potential heterogeneity.

#### Subgroup analysis by QoL instrument

**Fig. 4:**
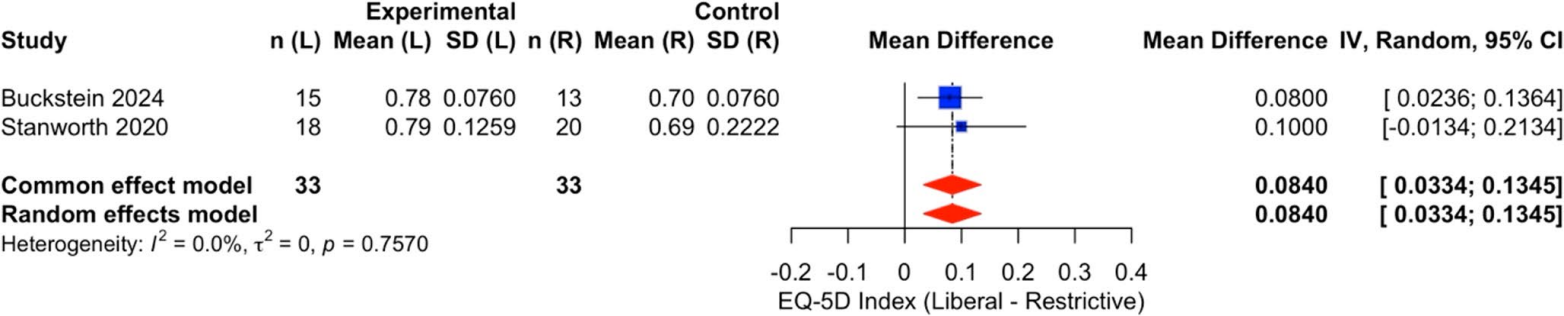
Forest plot summarising the subgroup analysis of mean difference in quality-of-life EQ-5D index scores between liberal transfusion and restrictive transfusion recipient groups across two studies (Buckstein 2024 and Stanworth 2020) [21, 22] with individual study effects as blue squares and pooled estimates as red diamonds. The pooled mean difference favours the liberal strategy with no statistical heterogeneity ($\:{I}^{2}=0\mathrm{\%}$, random effects model)

In the study by Buckstein et al. [21], EQ‑5D‑3 L pre‑transfusion means were reported; arm SDs were not reported. Hedges g derived from the reconstructed two-sample t-statistic for the between-arm difference (two-sided *p* = 0.01, df ≈ 26). The formula d = t·√(1/n_L + 1/n_R) was applied to convert the t-statistic to a standardized mean difference with Hedges correction. Stanworth et al. [22] measured QoL using EQ‑5D‑5 L single area under the curve (SAUC) medians (IQR) by arm over 12 weeks. For approximation, medians were treated as means and SD estimated from IQR via SD ≈ IQR/1.35; and Hedges g computed from these approximations. Temple et al. [23] reported EuroQol visual analogue scale (VAS) scores (from 0–100) at specified intervals, providing means and SDs. For consistency across studies, the 3-month timepoint was selected as closest to the typical 12-week endpoint. The forest plot in Fig. 4 summarises the subgroup analysis of mean difference in QoL.

Subgroup analysis of mean difference in EQ-5D index quality-of-life scores comparing liberal versus restrictive transfusion strategies across two studies (Buckstein 2024 and Stanworth 2020) [21, 22]. The pooled mean difference (MD) is 0.084 (95% CI: 0.033 to 0.134) (Fig. 4), favouring the liberal group with no observed heterogeneity ($\:{I}^{2}=0\mathrm{\%}$). Clinically, this suggests a small but statistically significant improvement in patient-reported quality of life with a liberal transfusion strategy, though the magnitude of benefit may be of limited practical significance in the context of clinical decision-making.

### II) Analysis of secondary outcomes

#### IIA) pooled mean difference in red blood cell (RBC) units transfused

**Fig. 5:**
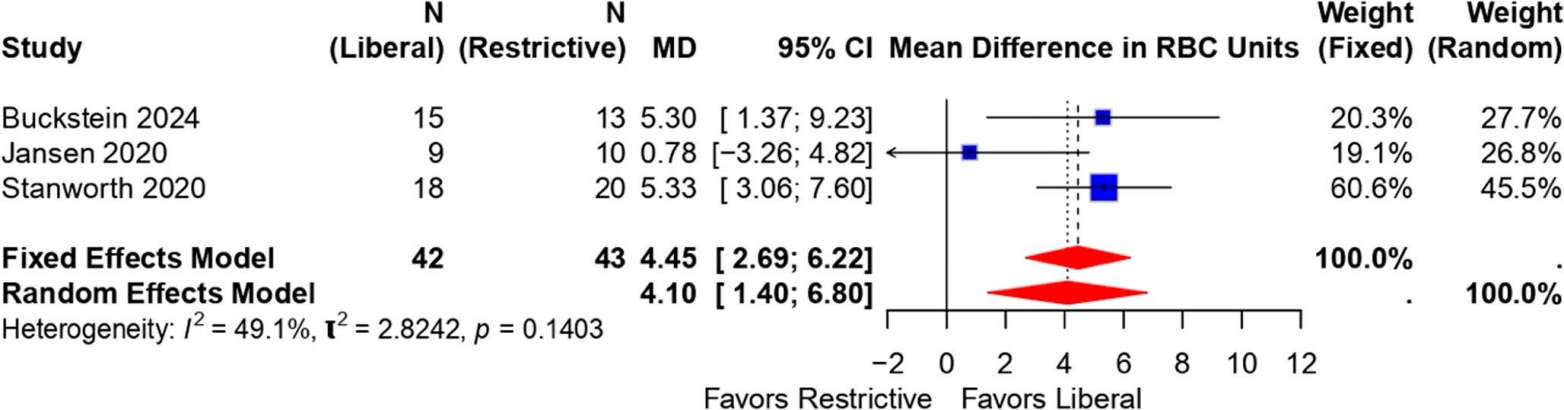
Forest plot of the mean difference in red blood cell units transfused per patient between liberal and restrictive transfusion recipient groups across three studies. Boxes represent the weighted mean difference for each study; horizontal lines represent 95% confidence intervals. The red diamond indicates the overall pooled mean difference estimate using a random-effects model

For Temple study by Jansen et al. [23] there were no SD data provided in the manuscript. So, it was imputed. For the REEDS study by Stanworth et al. [22] the median with IQR data was converted to mean and SD. Figure 5 forest plot demonstrated that liberal transfusion is associated with a significantly higher mean number of RBC units transfused per patient (random effects pooled mean difference 4.11 units; 95% CI 1.43 to 6.79). Moderate heterogeneity was observed (I² = 49.1%, *p* = 0.14).

#### Subgroup analysis of mean number of red cell units transfused per patient

**Fig. 6:**
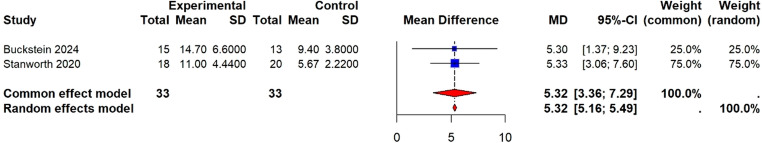
Subgroup analysis forest plot of the mean difference in red cell units transfused per patient between liberal and restrictive transfusion strategies in two trials. Blue squares represent individual study effects; the red diamond shows the pooled mean difference estimate

Subgroup meta-analysis of two randomized controlled trials (Stanworth 2020 and Buckstein 2024) [21, 22] demonstrated that liberal transfusion strategies resulted in a significantly greater mean number of red cell units transfused per patient compared to restrictive strategies (pooled mean difference 5.32 units; 95% CI 3.36 to 7.29) (Fig. 6). There was no evidence of statistical heterogeneity (I² = 0%).

##### IIB) Analysis of mortality hazard ratio

**Fig. 7:**
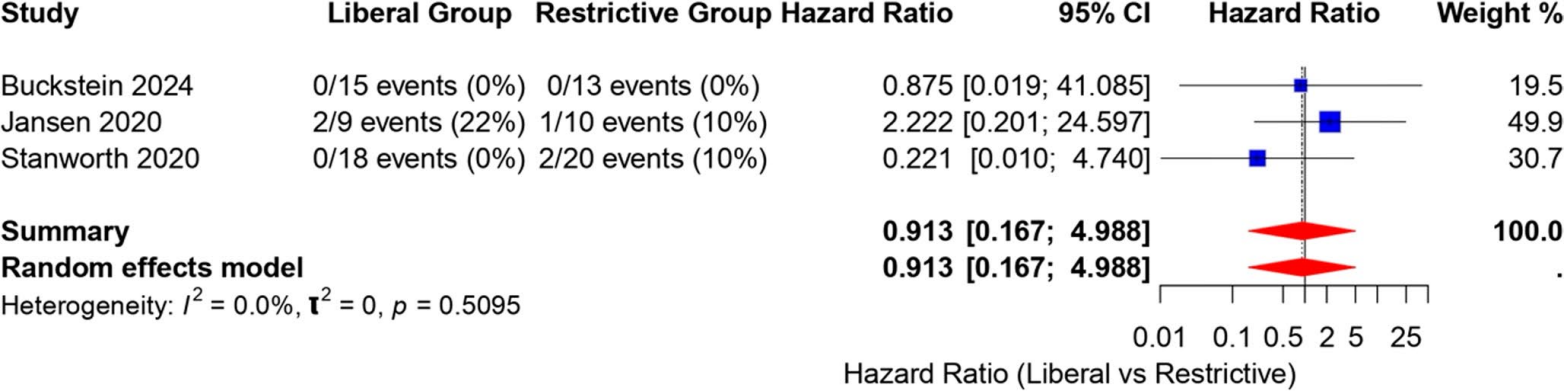
Forest plot illustrating the hazard in overall mortality between liberal transfusion and restrictive transfusion recipient groups for three studies [21–23]. The hazard ratio (HR) and corresponding 95% confidence intervals (CI) are displayed for each study, with the size of the blue squares proportional to the study weight. The pooled estimate from the random effects model is represented by a diamond, with its width indicating the 95% CI. Heterogeneity among studies is low (I² = 0%)

It was observed that overall pooled hazard ratio is 0.913 (95% CI: 0.167 to 4.98) (Fig. 7), indicating no significant difference in overall mortality between groups.

##### IIC) Analysis of transfusion reactions

**Fig. 8:**
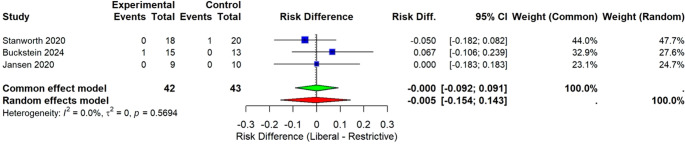
Forest plot depicting the risk difference for transfusion reaction rates across three studies [21–23]. Each study’s point estimate (RD) and 95% confidence interval (CI) are presented, with the square size reflecting the study’s weighting in the meta-analysis. The pooled estimate from the random effects model is illustrated by a diamond, whose width represents the 95% CI. Study heterogeneity is low (I² = 0%)

It was observed in RBC-ENHANCE trial (21) that patients in liberal arm had one episode of allo-immunization and one febrile nonhemolytic transfusion reaction. No reaction observed in the restrictive arm. The Temple and REDDS studies (22, 23) reported no adverse transfusion reactions in either the liberal or restrictive transfusion arms, indicating similar safety profiles between the two strategies in these trials. The overall pooled risk difference is−0.01 (95% CI: −0.10 to 0.09), indicating no statistically significant difference in transfusion reactions between the groups as shown in Fig 8.

##### IID) Analysis of rise in ferritin level (Fig. 9)

**Fig. 9:**
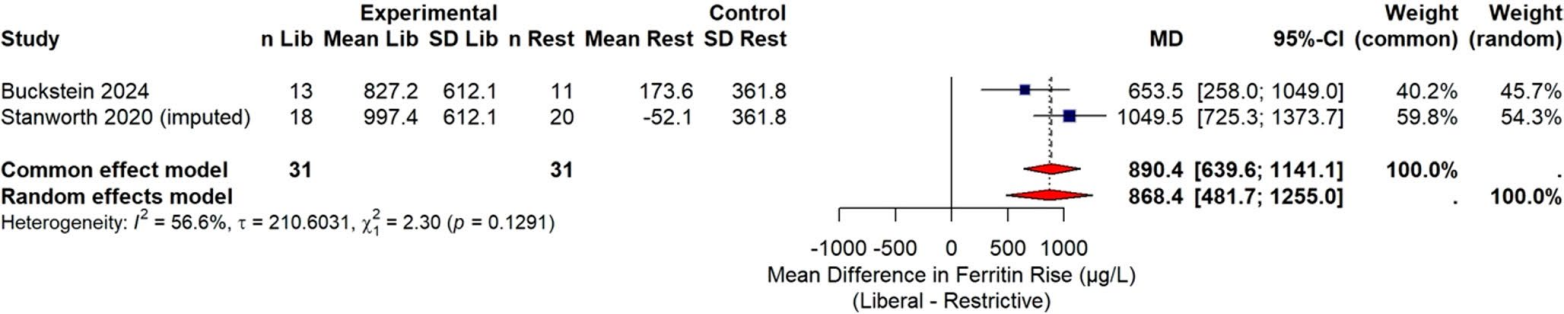
Forest plot of the absolute mean difference in ferritin rise (µg/L) between liberal and restrictive transfusion strategies in two studies [21, 22]. Buckstein 2024 [21] values are directly observed from patient-level data. Stanworth 2020 [22] values are imputed using participant-weighted back-calculation from pooled arm means and SDs borrowed from Buckstein 2024 [21], due to lack of reported arm-level summary statistics of ferritin level. Each row shows study arm sample sizes, mean and standard deviation of ferritin rise, and the associated mean difference (MD) with 95% confidence interval. The right panel displays the summary pooled effect for both the common (fixed) effect and random effects models, with heterogeneity statistics (I², τ)

## Discussion

The main objective of conducting this systematic review and meta-analysis was to compare safety and clinical outcomes of a liberal blood transfusion strategy and a restrictive blood transfusion strategy for patients with myelodysplastic syndrome who are not eligible or do not have access to haematopoietic stem cell transplant. The certainty of evidence for the whole population was low to moderate.

**Fig. 10:**
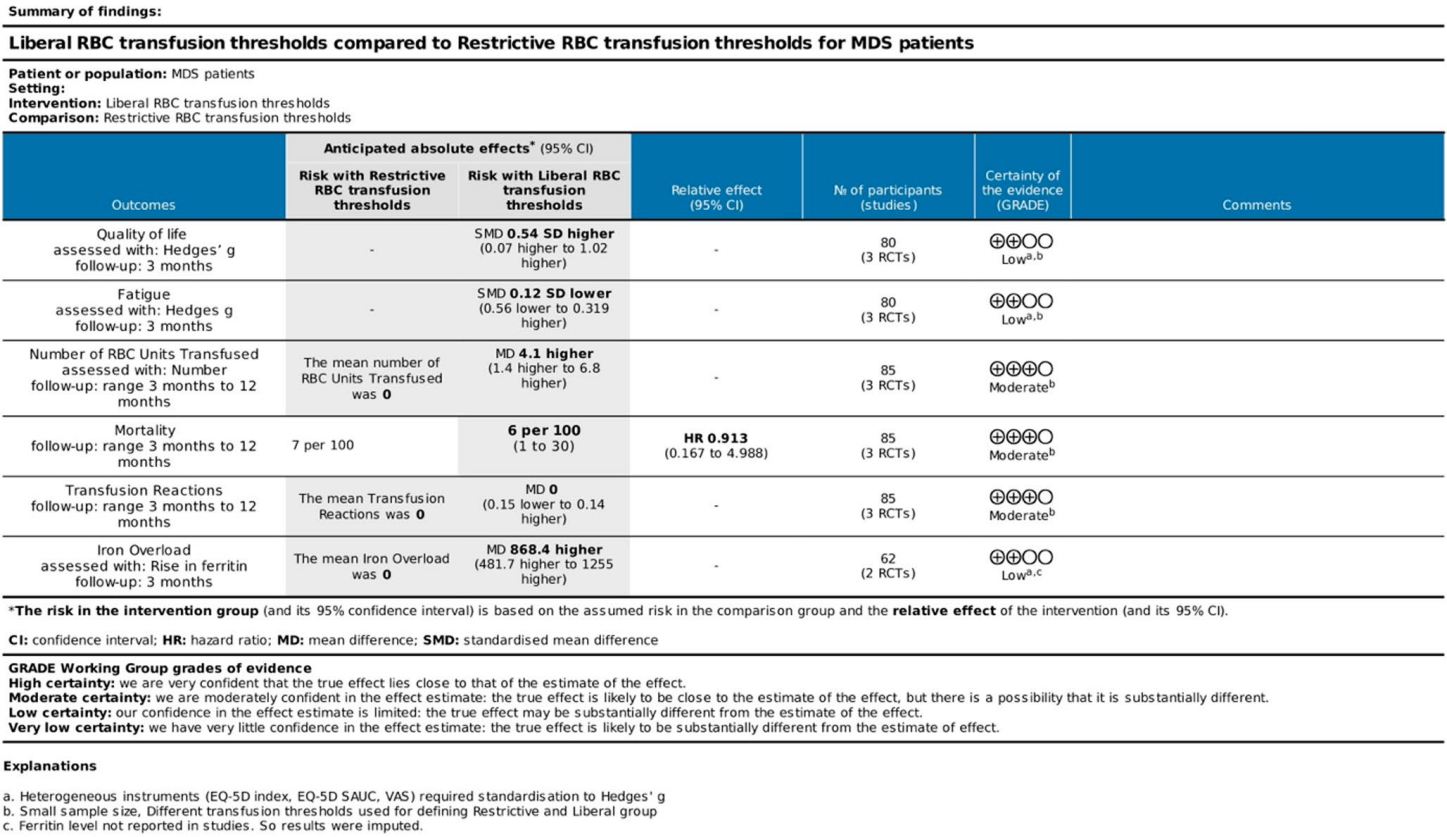
GRADE Summary of Findings comparing of safety and efficacy of liberal versus restrictive RBC transfusion thresholds on the quality of life in patients with MDS

In three small, randomised studies of patients with MDS who need transfusions, using a liberal transfusion strategy (keeping haemoglobin targets ~ 110–125 g/L or trigger < 9.7 g/dL) led to better patient-reported quality of life compared to a restrictive strategy (with lower haemoglobin targets ~ 85–105 g/L or trigger < 7.3 g/dL). The combined effect showed a moderate improvement in quality of life (Hedges g of 0.54) over 3 months period and possibly modestly lower fatigue (Hedges g of -0.12; lower is better; Supplementary Fig. 1). However, the overall confidence in this evidence is low due to limitations like small study sizes, differences in quality-of-life measures used, some study design issues (two feasibility/pilot trials and one prematurely terminated; clinicians unblinded) [21–23], concerns of bias and imprecision (small total N and wide CI) in the results. Only 1 study [23] included 12 months of follow up and data consistently shows better patient reported quality of life and less fatigue in the liberal transfusion strategy group (Supplementary Fig. 2). Despite these limitations, all studies consistently showed better quality of life with liberal transfusion. Subgroup analysis of studies reporting EQ-5D index scores was performed due to clinical heterogeneity of Hb threshold reported in different studies and tools used to report quality of life. The pooled mean difference of 0.084 (95% CI: 0.033 to 0.134) favours the liberal transfusion strategy, with no observed heterogeneity. This indicates a small but statistically significant improvement in quality-of-life, though the real-world clinical impact is likely minimal.

For RBC utilisation, patients in the liberal transfusion recipients received a significantly higher mean number of RBC units per patient (pooled mean difference 4.11 units; 95% CI 1.43–6.79), with moderate heterogeneity (I² = 48.7%). Subgroup meta-analysis of two RCTs further confirmed that liberal strategies led to increased transfusion requirements (pooled mean difference 5.32 units; 95% CI 3.36–7.29), without notable statistical heterogeneity.

Accumulation of iron stores far beyond physiological needs result in tissue damage and organ dysfunction mostly of heart, liver and endocrine organs. Liver fibrosis, liver failure, endocrine dysfunctions such as diabetes, hypothyroidism, hypoparathyroidism, Cardiac siderosis, left ventricular heart failure are complications of persistent iron overload [24, 25]. The current meta-analysis reinforces that liberal strategies while improving quality of life significantly increases cumulative iron intake. In this study we noted a significant rise in ferritin level in the liberal transfusion group than the restrictive group. In a recent study by Buckstein et al. [26] which pooled two trials REDDS and RBC-ENHANCE [21, 22], it was observed that mean ferritin levels were similarly elevated at baseline in both groups. However, over time, increased significantly more in the liberal transfusion arm by 926 µg/L (95% CI: 1446 to 2095), compared to a modest increase of 28 µg/L (95% CI: -864 to 922) in the restrictive arm, indicating a substantially higher risk of iron accumulation with liberal transfusion. They also reported higher inter-transfusion intervals in the restrictive arm (18.9 ± 9.9 days) than in liberal arm (13 ± 7.5 days). This means liberal group had frequent hospital visit than restrictive blood transfusion recipients.

Liberal blood transfusion does not enhance the overall mortality rate; pooled hazard ratio is 0.913 (95% CI: 0.167 to 4.98) with negligible heterogeneity (I² = 0%). There was no evidence of increased risk of transfusion reaction related risk in the liberal transfusion recipient group (pooled risk difference − 0.01, 95% CI − 0.10 to 0.09), though they received 5 units of higher blood transfusion than restrictive transfusion recipients. Certainly, recipients of blood transfusion as per liberal strategy did not experience extra short-term hazards related to transfusion than the restrictive group.

Liberal transfusion recipients on short term (12 weeks) to medium term (3 to 12 months) follow up did not have extra risk of transfusion reaction and mortality. However, they had received around extra 4–5 units of red blood transfusion and higher rise of ferritin level than the patients who were followed with restrictive blood transfusion threshold. Though liberal transfusion threshold had better patient reported better quality of life, based on the limited evidence, short follow up period and concerns for iron overload, this systematic review and meta-analysis could not recommend a liberal transfusion threshold for all MDS patients. Quality of life among MDS patients is not only dependent on Hb level [27]. So, the decision for blood transfusion threshold for MDS patients must be based on patient centred and multidisciplinary team approach involving medicine, haematology, oncology, physical medicine and rehabilitation, nursing staff, occupational therapist and transfusion specialists may be beneficial [3, 7, 28, 29].

The life expectancy of high and very high risk MDS patients, categorised as per IPSS-R [2] are limited to less than 1 year in majority patients [2, 6]. Whereas low risk and very low risk groups are managed long term with ESAs and have longer life expectancy. For very low risk and low risk MDS patients, the goal is to reduce transfusion dependence and to preserve quality of life while avoiding transfusion related complications [3, 4]. Adopting a restrictive transfusion strategy in very low risk and low risk MDS patients’ group can mitigate long term complications. However, this decision must be framed within multidisciplinary team discussion based on patient reported quality of life, balancing symptomatic benefit rather than following the strict Hb threshold. Liberal transfusion strategies appear to provide better improvement in quality of life in the short-term probably by maintaining higher haemoglobin levels that alleviate anaemia related symptoms and enhance functional status without much hazard of mortality and transfusion reaction. So, this strategy may be particularly helpful for patients at end-of-life and shorter life expectancy such as conservatively managed high-risk and very high risk MDS patients.

Across resource-limited settings in Asia, South America, and Africa, MDS transfusion support is constrained by limited blood supply, infrastructure gaps, inconsistent screening, and scarce access to chelation, heightening risks of iron overload and transfusion-transmitted infections [30–32] despite transfusions’ essential role in relieving symptomatic anaemia and improving quality of life. Equity-focused, context-specific guidance should balance benefits against risks and capacity, considering a liberal approach for patients with limited life expectancy after counselling on long-term harms while favouring a restrictive strategy for others to minimise iron burden and infectious risk.

### Future prospect

Greater certainty about the safety and efficacy of liberal red cell transfusion threshold on quality-of-life outcomes will require rigorously designed, adequately powered randomised controlled trials with long-term follow-up that report outcomes by established MDS risk strata [33]. Current evidence is insufficient to permit IPSS‑R based subgroup analyses in this systematic review and meta-analysis.

### Limitation

This review is limited by the small sample sizes of the included studies, which reduces the statistical power to detect differences and increases uncertainty around effect estimates. The limited number of participants also restricts the ability to perform robust subgroup analyses to explore potential effect modifiers. Additionally, there is considerable heterogeneity in the definitions of liberal and restrictive transfusion thresholds, QoL instruments across studies which contribute to challenges in directly comparing results and synthesizing findings. This inconsistency may affect the generalisability of the conclusions and underscores the need for standardised transfusion criteria in future research.

## Conclusion

In summary, it is difficult to reach a definitive conclusion given the relatively few included studies, low number of included participants, heterogeneity of intervention, and overall uncertainty of evidence. Liberal transfusion strategies in MDS patients appeared to improve patient reported quality of life with the trade-off of substantially increased transfusion requirements but did not significantly alter mortality or transfusion-related adverse event rates on short term follow up. Study heterogeneity for safety outcomes was minimal across meta-analyses. However, there are limited assessment of long-term consequences such as impact of iron overload or increased transfusion burden on cardiac remodelling, mortality, or other adverse effects in both transfusion arms. Based on the limited current evidence, a patient centric multidisciplinary team approach tailored to individual needs is essential to develop. To increase the certainty of safety and efficacy of a liberal red cell transfusion strategy on quality-of-life outcomes, there is a need for rigorously designed and executed studies specifically randomised controlled trials in larger populations with long term follow up.

## Supplementary information

Below is the link to the electronic supplementary material.

Supplementary Material 1

## Data Availability

Data will be available on request.

## References

[1] Garcia-Manero G (2023) Myelodysplastic syndromes: 2023 update on diagnosis, risk-stratification, and management. Am J Hematol 98(8):1307–1325. 10.1002/ajh.26984 37288607 10.1002/ajh.26984 PMC12002404

[2] Bernard E, Tuechler H, Greenberg PL, Hasserjian RP, Arango Ossa JE, Nannya Y, Devlin SM, Creignou M, Pinel P, Monnier L, Gundem G, Medina-Martinez JS, Domenico D, Jädersten M, Germing U, Sanz G, van de Loosdrecht AA, Kosmider O, Follo MY, Thol F, Zamora L, Pinheiro RF, Pellagatti A, Elias HK, Haase D, Ganster C, Ades L, Tobiasson M, Palomo L, Della Porta MG, Takaori-Kondo A, Ishikawa T, Chiba S, Kasahara S, Miyazaki Y, Viale A, Huberman K, Fenaux P, Belickova M, Savona MR, Klimek VM, Santos FPS, Boultwood J, Kotsianidis I, Santini V, Solé F, Platzbecker U, Heuser M, Valent P, Ohyashiki K, Finelli C, Voso MT, Shih LY, Fontenay M, Jansen JH, Cervera J, Gattermann N, Ebert BL, Bejar R, Malcovati L, Cazzola M, Ogawa S, Hellström-Lindberg E, Papaemmanuil E (2022) Molecular International Prognostic Scoring System for Myelodysplastic Syndromes. NEJM Evid 1(7):EVIDoa2200008. 10.1056/EVIDoa2200008 38319256 10.1056/EVIDoa2200008

[3] Killick SB, Ingram W, Culligan D, Enright H, Kell J, Payne EM, Krishnamurthy P, Kulasekararaj A, Raghavan M, Stanworth SJ, Green S, Mufti G, Quek L, Cargo C, Jones GL, Mills J, Sternberg A, Wiseman DH, Bowen D (2021) British Society for Haematology guidelines for the management of adult myelodysplastic syndromes. Br J Haematol 194(2):267–281. 10.1111/bjh.17612 34180045 10.1111/bjh.17612

[4] Fenaux P, Haase D, Santini V, Sanz GF, Platzbecker U, Mey U, ESMO Guidelines Committee (2021) Myelodysplastic syndromes: ESMO clinical practice guidelines for diagnosis, treatment and follow-up<Superscript>†☆</Superscript>. Ann Oncol 32(2):142–156. 10.1016/j.annonc.2020.11.002 33221366 10.1016/j.annonc.2020.11.002

[5] Platzbecker U (2019) Treatment of mds. Blood 7(10):1096–1107. 10.1182/blood-2018-10-844696 10.1182/blood-2018-10-844696 30670446

[6] Moreno Berggren D, Folkvaljon Y, Engvall M, Sundberg J, Lambe M, Antunovic P, Garelius H, Lorenz F, Nilsson L, Rasmussen B, Lehmann S, Hellström-Lindberg E, Jädersten M, Ejerblad E (2018) Prognostic scoring systems for myelodysplastic syndromes (MDS) in a population-based setting: a report from the Swedish MDS register. Br J Haematol 181(5):614–627. 10.1111/bjh.15243 29707769 10.1111/bjh.15243

[7] Balitsky A, Arnold D (2022) Transfusion thresholds in myelodysplastic syndrome-helping patients live better. Transfusion 62(7):1313–1314. 10.1111/trf.16985 35718934 10.1111/trf.16985

[8] Estcourt LJ, Malouf R, Trivella M, Fergusson DA, Hopewell S, Murphy MF (2017) Restrictive versus Liberal red blood cell transfusion strategies for people with haematological malignancies treated with intensive chemotherapy or radiotherapy, or both, with or without Haematopoietic stem cell support. Cochrane Database Syst Reviews ;(1):CD011305 10.1002/14651858.CD011305.pub2 PMC5298168 28128441

[9] Stauder R, Yu G, Koinig KA et al (2018) Health-related quality of life in lower-risk MDS patients compared with age- and sex-matched reference populations: a European LeukemiaNet study. Leukemia 32:1380–1392 29572506 10.1038/s41375-018-0089-x PMC5990524

[10] Haring Y, Goldschmidt N, Taha S et al (2023) MDS-related anemia is associated with impaired quality of life but improvement is not always achieved by increased hemoglobin level. J Clin Med 12(18):5865 37762806 10.3390/jcm12185865 PMC10532166

[11] Jansen AJ, Essink-Bot ML, Beckers EA et al (2003) Quality of life measurement in patients with transfusion-dependent myelodysplastic syndromes. Br J Haematol 121(2):270–274 12694248 10.1046/j.1365-2141.2003.04272.x

[12] Kracalik I, Mowla S, Basavaraju SV, Sapiano MRP (2021) Transfusion-related adverse reactions: data from the National healthcare safety network hemovigilance module – United States, 2013–2018. Transfusion 61(5):1424–1434 33880771 10.1111/trf.16362

[13] Gu Y, Estcourt LJ, Doree C, Hopewell S, Vyas P Restrictive versus liberal red cell transfusion for patients with myelodysplastic syndromes, aplastic I, and other congenital bone marrow failure disorders. *Cochrane Database of Systematic Reviews* 2015, Issue 10. Art. No.: CD011745. 10.1002/14651858.CD011745.pub2

[14] Page MJ, McKenzie JE, Bossuyt PM, Boutron I, Hoffmann TC, Mulrow CD, Shamseer L, Tetzlaff JM, Akl EA, Brennan SE, Chou R, Glanville J, Grimshaw JM, Hróbjartsson A, Lalu MM, Li T, Loder EW, Mayo-Wilson E, McDonald S, McGuinness LA, Stewart LA, Thomas J, Tricco AC, Welch VA, Whiting P, Moher D (2021) The PRISMA 2020 statement: an updated guideline for reporting systematic reviews. Syst Rev 10(1):89. 10.1186/s13643-021-01626-4 33781348 10.1186/s13643-021-01626-4 PMC8008539

[15] Higgins JPT, Thomas J, Chandler J, Cumpston M, Li T, Page MJ, Welch VA (eds) (2019) *Cochrane Handbook for Systematic Reviews of Interventions*. 2nd Edition. Chichester (UK): John Wiley & Sons

[16] Haddaway NR, Page MJ, Pritchard CC, McGuinness LA (2022) PRISMA2020: an R package and Shiny app for producing PRISMA 2020-compliant flow diagrams, with interactivity for optimised digital transparency and open synthesis Campbell systematic reviews. 18:e1230. 10.1002/cl2.1230. 10.1002/cl2.1230 PMC8958186 36911350

[17] Sterne JAC, Savović J, Page MJ, Elbers RG, Blencowe NS, Boutron I et al (2019) RoB 2: a revised tool for assessing risk of bias in randomised trials. BMJ 366:l4898 31462531 10.1136/bmj.l4898

[18] Parmar MKB, Torri V, Stewart L (1998) Extracting summary statistics to perform meta-analyses of the published literature for survival endpoints. Stat Med 17:2815–34 9921604 10.1002/(sici)1097-0258(19981230)17:24<2815::aid-sim110>3.0.co;2-8

[19] Tierney JF, Stewart LA, Ghersi D et al (2007) Practical methods for incorporating summary time-to-event data into meta-analysis. Trials 8:16. 10.1186/1745-6215-8-16 17555582 10.1186/1745-6215-8-16 PMC1920534

[20] McGuinness LA, Higgins JPT (2020) Risk-of-bias visualization (robvis): an R package and Shiny web app for visualizing risk-of-bias assessments. Res Syn Meth 1–7. 10.1002/jrsm.1411 10.1002/jrsm.1411 32336025

[21] Buckstein R, Callum J, Prica A, Bowen D, Wells RA, Leber B, Heddle N, Chodirker L, Cheung M, Mozessohn L, Yee K, Gallagher J, Parmentier A, Jamula E, Zhang L, Mamedov A, Stanworth SJ, Lin Y (2024) Red cell transfusion thresholds in outpatients with myelodysplastic syndromes: results of a pilot randomized trial RBC-ENHANCE. Transfusion 64(2):223–235. 10.1111/trf.17721 38323704 10.1111/trf.17721

[22] Stanworth SJ, Killick S, McQuilten ZK, Karakantza M, Weinkove R, Smethurst H, Pankhurst LA, Hodge RL, Hopkins V, Thomas HL, Deary AJ, Callum J, Lin Y, Wood EM, Buckstein R, Bowen D (2020) Red cell transfusion in outpatients with myelodysplastic syndromes: a feasibility and exploratory randomised trial. Br J Haematol 189(2):279–290. 10.1111/bjh.16347 31960409 10.1111/bjh.16347

[23] Jansen AJG, van den Bosch J, Te Boekhorst PAW, Schipperus MR, Beckers EAM (2020) Results of the prematurely terminated TEMPLE randomized controlled trial in patients with myelodysplastic syndrome: liberal versus restrictive red blood cell transfusion threshold. Transfusion 60(4):879–881. 10.1111/trf.15708 32246478 10.1111/trf.15708

[24] Taher AT, Saliba AN (2017) Iron overload in thalassemia: different organs at different rates. Hematology Am Soc Hematol Educ Program 2017(1):265–271. 10.1182/asheducation-2017.1.265 29222265 10.1182/asheducation-2017.1.265 PMC6142532

[25] Fibach E, Rachmilewitz EA (2017) Iron overload in hematological disorders. Presse Med 46(12 Pt 2):e296–e305. 10.1016/j.lpm.2017.10.007 29174474 10.1016/j.lpm.2017.10.007

[26] Buckstein R, Callum J, Prica A, Bowen D, Wells RA, Leber B, Heddle N, Chodirker L, Cheung M, Mozessohn L, Yee K, Gallagher J, Parmentier A, Jamula E, McQuilten Z, Wood EM, Weinkov R, Zhang L, Mamedov A, Stanworth SJ, Lin Y (2024) Red cell transfusion thresholds in outpatients with myelodysplastic syndromes: combined results from two randomized controlled feasibility studies. Am J Hematol 99(3):473–476. 10.1002/ajh.27181 38126081 10.1002/ajh.27181

[27] Wouters HJCM, Conrads-Frank A, Koinig KA, Smith A, Yu G, de Witte T, Wolffenbuttel BHR, Huls G, Siebert U, Stauder R, van der Klauw MM, MDS-RIGHT partners (2021) The anemia-independent impact of myelodysplastic syndromes on health-related quality of life. Ann Hematol 100(12):2921–2932. 10.1007/s00277-021-04654-1 34476573 10.1007/s00277-021-04654-1 PMC8592948

[28] Franchini M, Zani M, Focosi D (2025) Restrictive versus liberal transfusion thresholds: lights and shadows. Blood Transfus 23(1):93–95. 10.2450/BloodTransfus.884 39621886 10.2450/BloodTransfus.884 PMC11841946

[29] Koutsavlis I (2016) Transfusion thresholds, quality of life, and current approaches in myelodysplastic syndromes. Anemia 2016:8494738. 10.1155/2016/8494738 27195147 10.1155/2016/8494738 PMC4853931

[30] Jenny HE, Saluja S, Sood R, Raykar N, Kataria R, Tongaonkar R et al (2017) Access to safe blood in low-income and middle-income countries: lessons from India. BMJ Global Health 2. 10.1136/bmjgh-2016-000167. :bmjgh-2016-000167 10.1136/bmjgh-2016-000167 PMC5584485 30206488

[31] Inusa BP, Atoyebi W, Andemariam B, Hourani JN, Omert L (2023) Global burden of transfusion in sickle cell disease. Transfus Apher Sci 62(5):103764. 10.1016/j.transci.2023.103764 37541800 10.1016/j.transci.2023.103764

[32] Custer B, Zou S, Glynn SA, Makani J, Tayou Tagny C, El Ekiaby M, Sabino EC, Choudhury N, Teo D, Nelson K, Peprah E, Price L, Engelgau MM (2018) Addressing gaps in international blood availability and transfusion safety in low- and middle-income countries: a NHLBI workshop. Transfusion 58(5):1307–1317. 10.1111/trf.14598 29542130 10.1111/trf.14598 PMC6510980

[33] Kaka S, Jahangirnia A, Beauregard N, Davis A, Tinmouth A, Chin-Yee N (2022) Red blood cell transfusion in myelodysplastic syndromes: a systematic review. Transfus Med 32(1):3–23. 10.1111/tme.12841 34927286 10.1111/tme.12841

