# Supplementary material for: Comparison of safety and efficacy of liberal versus restrictive red blood cell transfusion thresholds on the quality of life in patients with myelodysplastic syndromes: a systematic review and meta-analysis
Source: Ann Hematol. 2026 Jan 26;105(2):61. doi: 10.1007/s00277-026-06789-5 (PMC12835097; doi:10.1007/s00277-026-06789-5)
Supplement: Supplementary file 1 — Supplementary Material 1 [file 277_2026_6789_MOESM1_ESM.docx]

**Appendix – 1**

**Database: Ovid MEDLINE(R) ALL <1946 to August 8, 2025>**

Search Strategy:

1 exp Myelodysplastic Syndromes/ (25324)

2 myelodysplas*.mp. (29298)

3 "dysmyelopoietic syndrome*".mp. (55)

4 "refractory an?emia*".mp. (2798)

5 "sideroblastic an?emia*".mp. (1552)

6 ((gata2 or gata-2) adj1 (deficienc* or haploinsufficienc*)).mp. (276)

7 "paroxysmal h?emoglobinuria".mp. (151)

8 MDS.mp. (26457)

9 "refractory cytopenia*".mp. (424)

10 5q-syndrome*.mp. (339)

11 1 or 2 or 3 or 4 or 5 or 6 or 7 or 8 or 9 or 10 (49323)

12 exp Blood Transfusion/ (95839)

13 transfusion*.mp. (183934)

14 12 or 13 (191039)

15 11 and 14 (3328)

16 (threshold* or trigger* or "cut off*" or "decision point*" or "critical level*").mp. (921817)

17 15 and 16 (97)

**Database: Embase <1974 to 2025 Week 32>**

Search Strategy:

1 exp myelodysplastic syndrome/ (65258)

2 myelodysplas*.mp. (70259)

3 "dysmyelopoietic syndrome*".mp. (70)

4 "refractory an?emia*".mp. (8321)

5 "sideroblastic an?emia*".mp. (2526)

6 ((gata2 or gata-2) adj1 (deficienc* or haploinsufficienc*)).mp. (1047)

7 "paroxysmal h?emoglobinuria".mp. (116)

8 MDS.mp. (59122)

9 "refractory cytopenia*".mp. (2847)

10 5q-syndrome*.mp. (1214)

11 1 or 2 or 3 or 4 or 5 or 6 or 7 or 8 or 9 or 10 (103004)

12 exp transfusion/ (417538)

13 transfusion medicine/ (2767)

14 transfusion*.mp. (339984)

15 12 or 13 or 14 (498974)

16 11 and 15 (15229)

17 (threshold* or trigger* or "cut off*" or "decision point*" or "critical level*").mp. (1254875)

18 16 and 17 (642)

**Supplementary Table 1:** Systematic Review comparing Liberal and Restrictive RBC transfusion approaches in MDS patients for patient reported fatigue score (Hedges g)

| **Study** | **Scale** | **N Liberal** | **N Restrictive** | **Mean Liberal** | **SD Liberal** | **Mean Restrictive** | **SD Restrictive** | **Hedges g** | **SE** | **CI Lower** | **CI Upper** |
| --- | --- | --- | --- | --- | --- | --- | --- | --- | --- | --- | --- |
| Jansen 2020 | MFI | 7 | 7 | 10.3 | 5.2 | 11.7 | 5.7 | 0.24 | 0.538 | -0.811 | 1.292 |
| Stanworth 2020 | EORTC QLQ-C30 | 18 | 20 | 34 | 38.5 | 38 | 15.6 | 0.136 | 0.325 | -0.501 | 0.774 |
| Buckstein 2024 | EORTC QLQ-C30 | 15 | 13 | 28.06 | 14.29 | 28.7 | 17.18 | 0.04 | 0.379 | -0.703 | 0.782 |

**Supplementary Figure 1:**

**
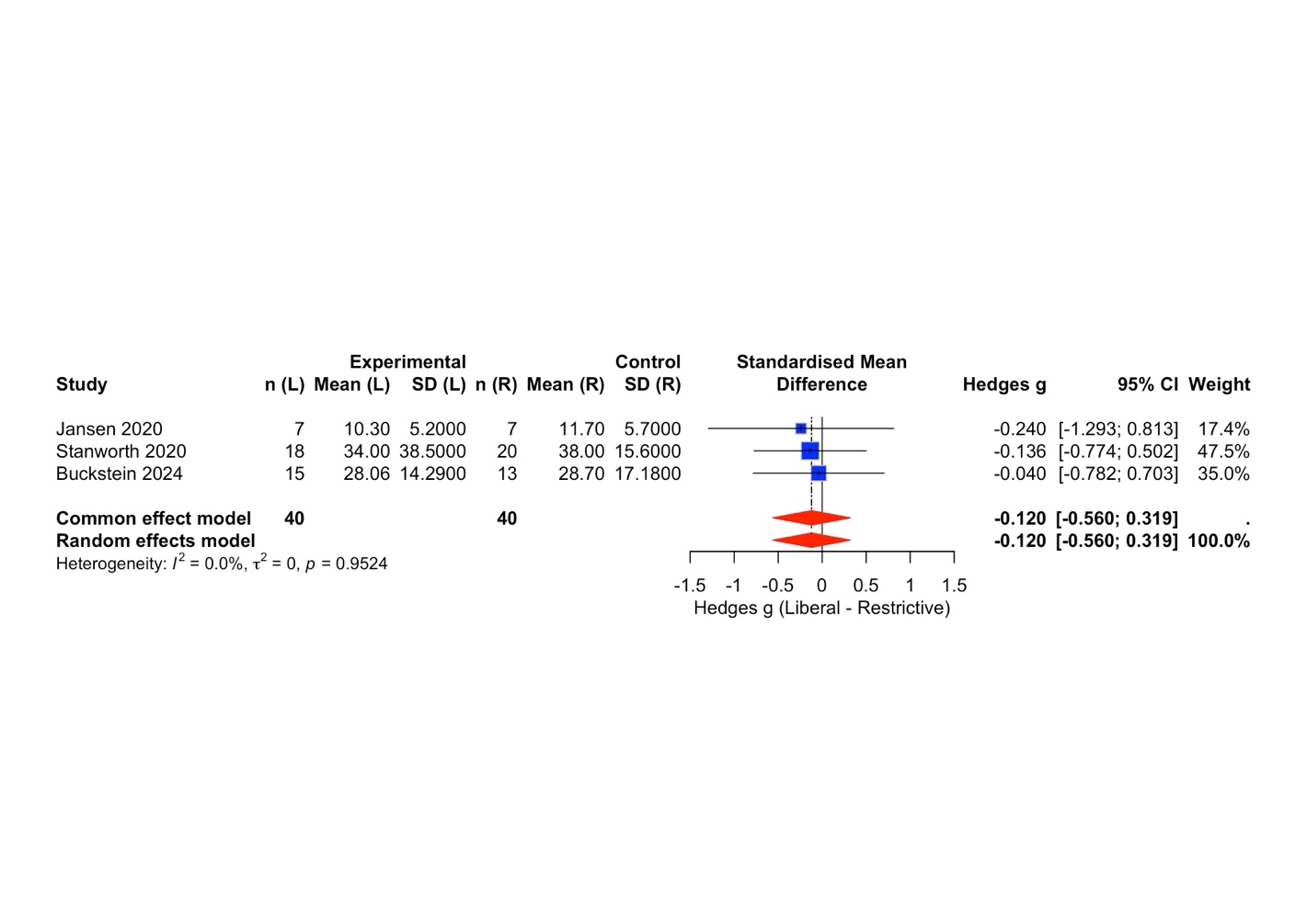
**

**Supplementary Figure 1:** Forest plot of standardized mean differences (Hedges g) in fatigue comparing liberal versus restrictive transfusion strategies across three studies (21, 22, 23), with random- and common-effects pooled estimates and heterogeneity statistics displayed. Each row shows study-level sample sizes, means, and standard deviations for experimental (liberal) and control (restrictive) arms, alongside the study’s standardized mean difference with 95% confidence interval and its weight under common and random-effects models. The red diamonds depict pooled effects from common- and random-effects models; both pooled estimates center near zero (SMD −0.12, 95% CI −0.56 to 0.32), indicating no statistically significant difference in fatigue between liberal and restrictive strategies.

Across three trials (21, 22, 23), fatigue scores were modestly lower with the liberal transfusion strategy, but study‑level effects were small and imprecise with confidence intervals crossing no difference. The pooled standardized mean difference was −0.12 (95% CI −0.56 to 0.32), where negative values favour the liberal arm because lower fatigue scores are better. Heterogeneity was negligible (I² = 0%, τ² = 0; p = 0.9524), indicating consistent findings across studies. Overall, evidence does not show a statistically significant advantage of either strategy for reducing fatigue, despite a slight trend toward lower fatigue with liberal transfusion.


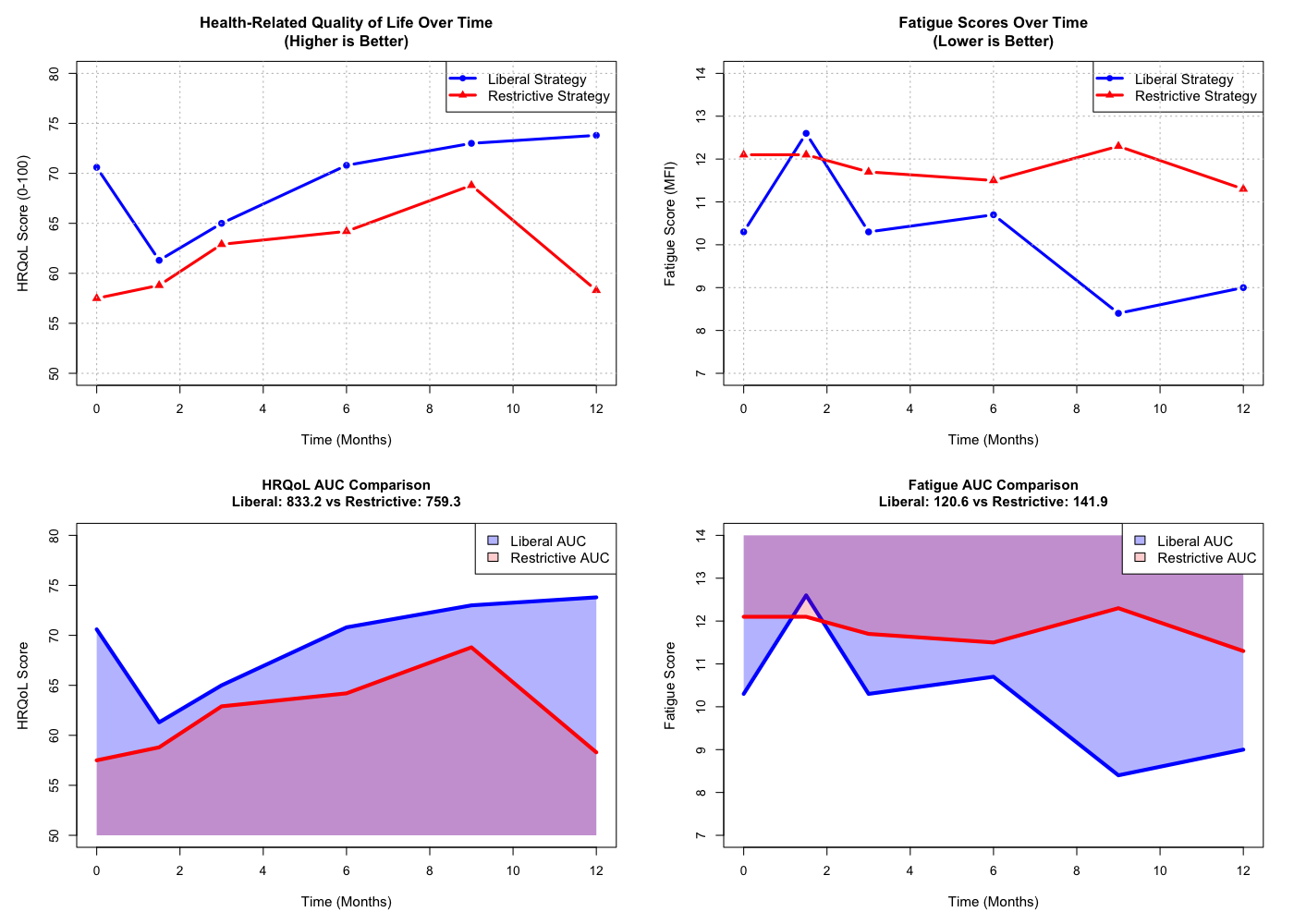


**Supplementary Figure 2:** Longitudinal health-related quality of life (HRQoL) and fatigue outcomes comparing liberal versus restrictive transfusion strategies over 12 months in adults with myelodysplastic syndromes.

- Panel A: HRQoL scores on a 0–100 scale (higher indicates better status) plotted at months 0, 2, 3, 6, 9, and 12 for liberal and restrictive strategies, with lines connecting group means over time.
- Panel B: Fatigue measured with the Multidimensional Fatigue Inventory (MFI), where lower scores indicate less fatigue, shown at the same timepoints for both strategies with mean trajectories.
- Panel C: Cumulative HRQoL expressed as area under the curve (AUC) across 12 months, displaying shaded areas and totals for each strategy (Liberal AUC 833.2 vs Restrictive AUC 759.3), where larger AUC reflects better overall HRQoL over time.
- Panel D: Cumulative fatigue AUC across 12 months with shaded areas and totals (Liberal 120.6 vs Restrictive 141.9), where a smaller AUC reflects lower overall fatigue burden over time.

Abbreviations: HRQoL, health-related quality of life; MFI, Multidimensional Fatigue Inventory; AUC, area under the curve.

**
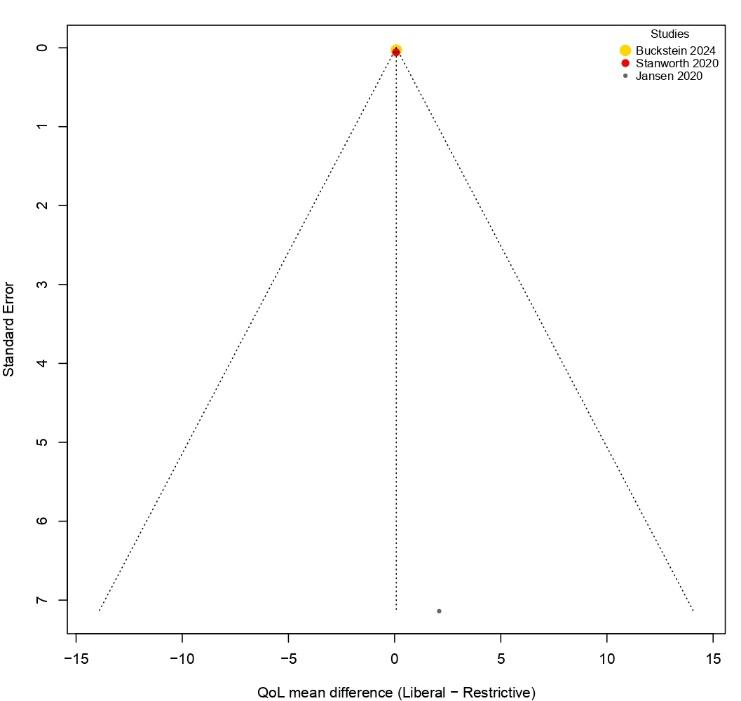
**

**Supplementary Figure 3:** Funnel plot shows QoL mean difference (Liberal-Restrictive transfusion strategy) for three studies. This funnel plot shows that most evidence comes from two large, precise studies (Buckstein 2024, Stanworth 2020) that both suggest little difference in QoL between transfusion strategies. The plot is symmetrical, but because only three studies are included, we cannot meaningfully evaluate publication bias
